# Nav1.7 via Promotion of ERK in the Trigeminal Ganglion Plays an Important Role in the Induction of Pulpitis Inflammatory Pain

**DOI:** 10.1155/2019/6973932

**Published:** 2019-03-28

**Authors:** Shukai Sun, Jiangxing Sun, Wenkai Jiang, Wei Wang, Longxing Ni

**Affiliations:** ^1^State Key Laboratory of Military Stomatology & National Clinical Research Center for Oral Diseases & Shaanxi Key Laboratory of Stomatology, Department of Operative Dentistry & Endodontics, School of Stomatology, Fourth Military Medical University, No. 145 Western Changle Road, Xi'an, Shaanxi 710032, China; ^2^Department of Ophthalmology, Eye Institute of Chinese PLA, Xijing Hospital, Fourth Military Medical University, China

## Abstract

The trigeminal ganglion (TG) refers to sensory neurons bodies that innervate the spinal cord and peripheral axons that innervate teeth. The tetrodotoxin-sensitive sodium (NA) channels (Nav1.7) play important roles in the pathophysiology of pain. In this study, we investigated the TG expression of Nav1.7 and extracellular signal-regulated kinase (ERK) in a rat model of pulpitis to explore the correlation between these channels and inflammatory pain. Pulpitis was confirmed by hematoxylin-eosin staining. In this study, we demonstrated that the reflex of rats to mechanical stimulation increases after pulp exposure and that the exposed rat molar pulp can upregulate the expression of Nav1.7 and ERK in the rat TG. Three days after rat pulp exposure, the expression levels of the two ion channels in the TG increased. TG target injection of PF04856264, a Nav1.7 inhibitor, dose-dependently increased the mechanical pain threshold and was able to inhibit ERK expression. TG target injection of PD98059, an ERK inhibitor, dose-dependently increased the mechanical pain threshold. These factors simultaneously resulted in the highest production. In this study, with the established link to inflammatory pain, we found that Nav1.7 and ERK both play important roles in the induction of inflammatory pain caused by pulpitis. We also found a correlation between the expression levels of Nav1.7 and ERK and the degree of inflammatory pain. Furthermore, ERK signaling pathways were promoted by the Nav1.7 in TG after pulpitis.

## 1. Introduction

The main cause of dental pain is due to pulpitis. The TG consists of sensory neuronal bodies, the central and peripheral axons of which innervate the spine and teeth, respectively [1]. Changes in primary afferent neurons result in increased excitability [2, 3], which induces the generation of hyperalgesia [4]. These functional changes must be regulated by specific signaling systems in neurons [5].

Voltage-gated sodium channels (VGSCs) govern the electrical excitability of peripheral nerves and play an important role in the pathophysiology of pain. VGSCs may be classified as fast-activating neurotoxin tetrodotoxin (TTX)-sensitive (Nav1.7) channels and slow-inactivating TTX-resistant channels (TTX-R) [6]. Nav1.7 is an important element of VGSC expression in peripheral nerves. In an inflammatory pain mouse model, the knockout of Nav1.7 revealed deficits in behavioral studies [7]. Nav1.7 in nociceptors plays a central role in skeletal muscle nociceptive sensitization [8].

It has been shown that the activation of mitogen-activated protein kinase (MAPK) signaling pathways plays an important role in the development and persistence of pain [9–11]. There are three major MAPK family members: ERK, p38, and c-Jun N-terminal kinase (JNK). ERK can modulate the activities of Nav1.7 [12]. However, no studies have examined the relationship between ERK and Nav1.7 in pulpitis. Dental pulp contains nerves that deliver the afferent input of TG neurons [13]. To the best of our knowledge, the expression levels of Nav1.7 and ERK in the trigeminal ganglion in a pulpitis model have not been studied prior to the present study. Thus, the purpose of this study was to determine the expression of Nav1.7 and ERK in the trigeminal ganglion in a rat model of pulpitis and to explore the relationship between ERK and Nav1.7 in the rat trigeminal ganglion in a model of pulpitis to aid the development of future treatments for painful dental diseases.

## 2. Methods and Methods

### 2.1. Animals

Sprague-Dawley (SD) male rats (220–250 g) were housed in a temperature-controlled room at 22–25°C with a 12-h light/12-h dark cycle. All rats were housed in plastic cages (six animals per cage) with food and water provided ad libitum. The animal experiments in this study were conducted with ethical approval by the Animal Use and Care Committee for Research and Education of the Fourth Military Medical University (permission number: SYXK2008-005). The rats were randomly assigned to six groups (six rats in each group): group 1 was sham-operated, groups 2–4 had their dental pulp exposed for 3, 7, and 14 days, and groups 5–6 had their dental pulp exposed for 14 days except that they were given PF04856264 or PD98059 at a dose of 10.0 *μ*M/L.

### 2.2. Tooth Pulp Exposure Procedure

The rats were deeply anesthetized through an intraperitoneal injection of pentobarbital (60 mg/kg) and fixed on a rat stereotaxic frame (Stoelting, Kiel, WI, USA). To stabilize the rats during the drilling procedure, their zygomatic arch was immobilized using a bar on each side of the head. The rats were positioned face up and their mouths were kept open using a rubber retractor fixed on the surgery platform and the rats' maxillary and mandibular incisors. To view the rats' molars, we performed the procedure using a surgical microscope (X500, Motic, Richmond, BC, Canada). The pulp of the rats' maxillary left first molar was exposed using a dental bur (with a #1 round tip) rotating slowly without any further treatment. The rats were then returned to their cages. The pulp of the rats in the sham-operated groups was not exposed and their teeth were kept intact; rats were anesthetized only. The rats were observed after the operation to ensure that they were not hurt due to pulp exposure, which may have resulted in a disruption in feeding and in a loss in weight. According to previously described protocol, we placed Fluorogold crystals (Fluorochrome, Englewood, CO, USA) into the cavity for the retrograde labeling of pulpal afferents. To prevent leakage, we sealed the cavity with a light-cured resin (Maxcem, Kerr, Orange, CA, USA).

### 2.3. TG Injections

We carried out the peripheral target injection to the TG via the infraorbital foramen, following procedures that Neubert et al. described [14]. Briefly, on postoperative day 14, rats were deeply anesthetized through an intraperitoneal injection of pentobarbital (60 mg/kg). A sterile stainless-steel needle was then inserted medially (1–2 mm) to the palpated portion of the zygomatic process through the infraorbital foramen. The needle was positioned at a 10-degree angle relative to the midline of the head. The tip of the needle was advanced approximately 20 mm along the infraorbital canal and subsequently through the foramen rotundum, and the reagents (5*μ*L) were slowly delivered to the trigeminal ganglion. The mechanical pain threshold was then determined every 1 h after injection.

### 2.4. Behavioral Testing

In the behavioral experiments, four groups of rats [the sham-operated (n = 9), the pulp exposure group (n = 9), the PF04856264 (n = 9) and PD98059 (n = 9)] were tested for their withdrawal threshold (WT) to mechanical stimulation of the facial skin. The rats were acclimatized to the test three days prior to baseline testing and were then placed in a small handmade porous metal mesh cage. During this period, a von Frey filament was slowly inserted into the mesh while the animal's head was resting steadily. Mechanical hypersensitivity was assessed using calibrated von Frey filaments (Stoelting, Kiel, WI, USA). The log stiffness of the filaments was determined based on log⁡10 (milligrams ×10). The filaments had the following log-stiffness values: 3.84, 4.08, 4.17, 4.31, 4.56, 4.74, 4.93, 5.07, 5.18, 5.46, and 5.88. Positive signs of withdrawal included pulling back rapidly, biting, and shaking the head within 5 s of being pricked by one of the von Frey filaments. The interval between trials was at least 3 min. For each trial, the same rat was stimulated 10 times by a single von Frey filament before being stimulated by the next largest filament. The WT recorded was the minimal value that resulted in at least six responses to ten stimulations.

### 2.5. Histology

The protocol was performed as previously described [15–17]. All paraffin-embedded samples were analyzed by hematoxylin-eosin (H-E) staining in this study. The animals were rapidly and deeply anesthetized through an intraperitoneal injection of pentobarbital (60 mg/kg) and sacrificed. The left dental pulp was fixed in 4% paraformaldehyde at 4°C overnight, followed by demineralization in 10% EDTA solution (Sigma) over 4 weeks and then dehydrated. The specimens were dehydrated in ethanol (70%, 95%, 100%, plus Xylol) and then put into paraffin molds to be sectioned with microtome at the level of the cement-enamel junction in 5 *μ*m slices for histological examination. The slices were maintained in a water bath at 40°C and dried in a furnace for one hour at 56°C. The slides were deparaffinized in xylene (2 × 5 min) and rehydrated with successive 1-min washes in 100%, 96%, 80%, and 70% ethanol. They were then stained with hematoxylin (2 min), rinsed with distilled water, rinsed with 0.1% hydrochloric acid in 50% ethanol, rinsed with tap water for 15 min, stained with eosin for 1 min, and rinsed again with distilled water. The slides were then dehydrated with 95% and 100% ethanol successively followed by xylene (2 × 5 min) and mounted with coverslips. Sections were examined using standard light microscopy.

### 2.6. Western Blotting Analyses

The animals were rapidly and deeply anesthetized through an intraperitoneal injection of pentobarbital (60 mg/kg) and sacrificed. The left trigeminal ganglion was dissected on ice and immediately stored at –80°C. A hand-held pestle was used to homogenize the TG in 4°C RIPA Lysis Buffer (Beyotime Biotechnology, China) with a mixture of proteinase and phosphatase inhibitors (Sigma, MO, USA). The protein concentrations were estimated using the bicinchoninic acid (BCA) assay. The samples were heated in boiling water for 5 min, loaded onto 5% stacking/12% separating gels (Bio-Rad Laboratories, CA, USA), and transferred onto a polyvinylidene difluoride membrane (PVDF, Immobilon-P, Millipore, Billerica, MA, USA). The membranes were blocked in 3% BSA solution for 2 h and probed with the following primary antibodies overnight at 4°C: rabbit anti-p-JNK (1:500, Millipore), rabbit anti-JNK (1:500, Millipore), mouse anti-Nav1.7 (1:1000, Sigma), and mouse anti-*β*-actin (1:3000, Sigma). The membranes were then incubated with the following secondary antibodies for 2 h: HRP-conjugated anti-rabbit (1:5000, Zhongshan, Beijing, China) and HRP-conjugated anti-mouse (1:5000, Zhongshan). The membranes were rinsed with Tris-buffered saline Tween-20 (TBST) three times (10 min each) between each step. The relative content of the target proteins was detected using enhanced chemiluminescence (ECL) and exposure to film.

### 2.7. Immunohistochemistry

The animals were deeply anesthetized through an intraperitoneal injection of pentobarbital (60 mg/kg) and perfused with phosphate-buffered saline (PBS, pH 7.2–7.4) and then with 4% paraformaldehyde in phosphate buffer (0.1 M, pH 7.4). The TG was harvested and immersed in 30% sucrose overnight at 4°C; 14 *μ*m frozen sections were then cut using a cryostat. The sections were rinsed with PBS (pH 7.2–7.4) three times (10 min each) and blocked for 1 h at 37°C with 0.01 M PBS containing 10% normal goat serum and 0.3% Triton X-100. The sections were then incubated for 48 h at 4°C with rabbit anti-FG (1:1000, Sigma) and mouse anti-Nav1.7 (1:1000, Sigma) [18–20]. After washing in PBS, the sections were incubated for 1 h with Alexa 488-conjugated donkey anti-mouse (secondary antibody) (1:500, Invitrogen, Eugene, OR, USA) and Alexa 594-conjugated donkey anti-mouse (secondary antibody) (1:500, Invitrogen, Eugene, OR, USA). Bicinchoninic acid images were captured with a confocal laser-scanning microscope (Olympus FV1000, Tokyo, Japan). To determine the number of neurons for Nav1.7, only neurons with clearly visible nuclei were counted. Neurons were determined as positive if the signal intensity was threefold higher than the background using Stereo Investigator software (MicroBrightField, Williston, VT, United States). The neurons were then counted in at least five randomly selected TG sections per animal [21].

### 2.8. Statistics

The data obtained from the behavioral studies passed the test for normality and were thus suitable for analysis using parametric statistics. To analyze the data, we used two-way ANOVA followed by Tukey's test (multiple groups) or t-test (two groups) for post hoc analysis. To analyze the western blot data, we used two-way ANOVA followed by the Newman-Keuls test for post hoc analysis. All of the statistical analyses were performed using the SPSS version 16.0 software. All of the data are presented as the mean ± SEM, and p<0.05 was considered to be statistically significant in all cases.

## 3. Results and Discussion

**Figure 1:**
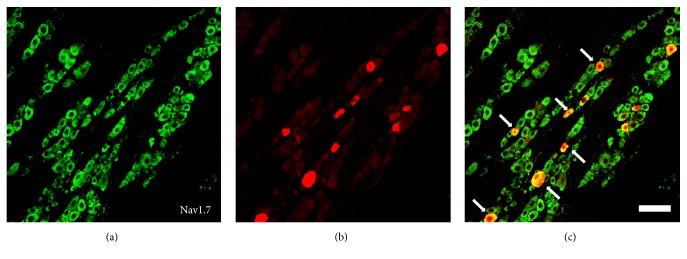
Expression of Nav1.7 in the TG pulpal afferent neurons. (a) Immunohistochemical staining with Nav1.7. (b) Fluorogold-labeled primary afferents in the TG section. (c) Merged image of (a) and (b). The arrow indicates a Nav1.7 positive pulpal afferent neuron. Scale bar: 100 *μ*m.

To investigate the possible involvement of pulpitis in the regulation of JNK and Nav1.7 expression, SD rats were subjected to normal tooth pulp, tooth pulp exposure for 3, 7, and 14 days, and Fluorogold was applied to retrogradely label the pulpal afferents. We used behavioral testing to determine pain reactions. We used western blotting and immunohistochemistry analyses to determine the expression of the JNK and Nav1.7 in TG. These were identified by the discrete gold-colored particles within the cytoplasm (Figure 1(b)). To demonstrate the expression of Nav1.7 in the rat pulpal afferents, the TG were identified using immunohistochemical staining and antibodies against Nav1.7 (Figure 1(a)). The expression of Nav1.7 is determined in the rat pulpal afferents because some neurons were colabeled using either Fluorogold and or Nav1.7 (Figure 1(c)).

### 3.1. Morphologic Changes in Dental Pulp

**Figure 2:**
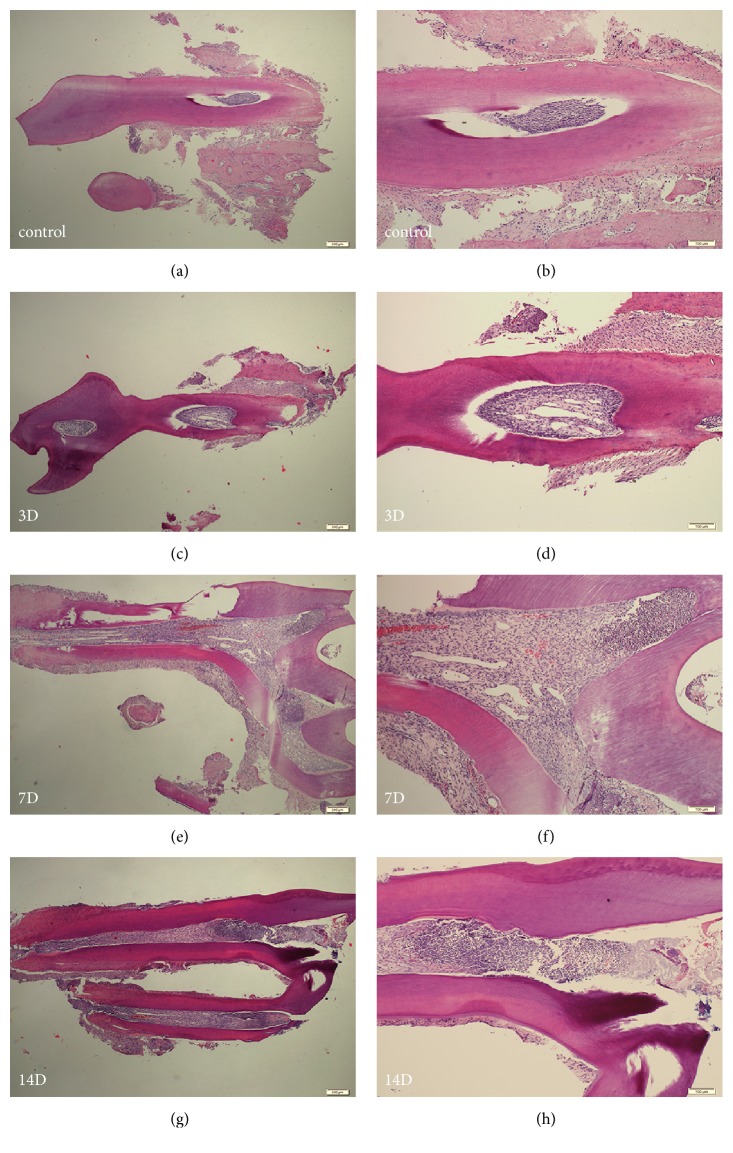
Hematoxylin-eosin staining of sagittal sections of left first maxillary molars. (a) Sham-operated rat at 4×magnification. (b) Sham-operated rat at 10×magnification. (c, e, g) The cavities were made, and acute inflamed dental pulps at 3, 7, and 14 days are shown at 4×magnification. (d, f, h) The cavities were made, and acute inflamed dental pulps at 3, 7, and 14 days are shown at 10×magnification.

To examine whether the dental cavities were prepared and dental pulp inflammation was induced successfully, we did H-E staining by using sections of the left maxillary incisor molars at 3, 7, and 14 days after the tooth pulp exposure procedure and for the sham-operated group. Our results indicated that dental cavities were induced successfully and inflammation was detectable as early as 3 days and was more severe at 7 and 14 days after the tooth pulp exposure procedure (Figure 2).

### 3.2. Pulp Exposure Increased Rat Reflex to Mechanical Stimulation

**Figure 3:**
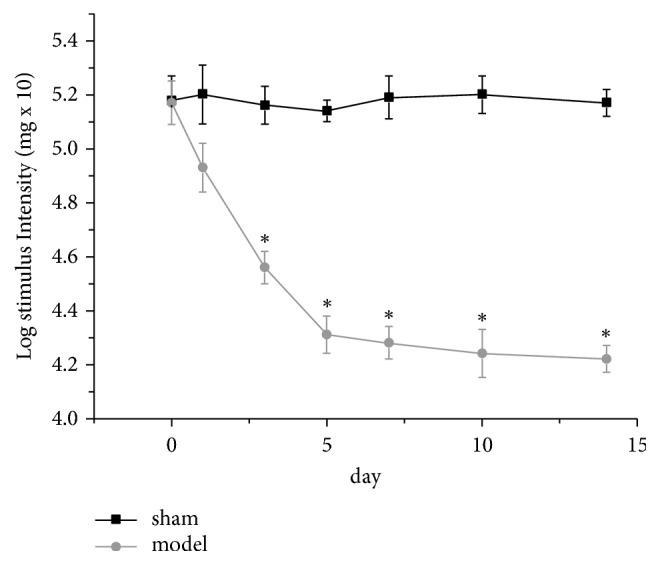
Effects of pulp exposure on mechanical hypersensitivity. The effects were studied starting one day prior to and 14 days after pulp exposure. Each point represents the mean ± SEM of the animals' withdrawal thresholds in response to mechanical stimuli. The experiment involves two groups (control and pulp-exposed groups), and each group included nine rats. *∗* indicates a statistically significant difference (p<0.05) between the two groups.

The mechanical pain threshold was determined one day before and every three days after the tooth pulp exposure procedure. The baseline and subsequent daily WTs in response to mechanical stimulation of the mandibular skin of the intact rats were approximately 5.18 (Figure 3). The WTs were reduced to approximately 4.56 within three days after pulp exposure compared with the baseline WTs. This reduction was significant after five days and lasted for at least 14 days.

### 3.3. Expression of Nav1.7 in a Rat Model of Pulpitis

**Figure 4:**
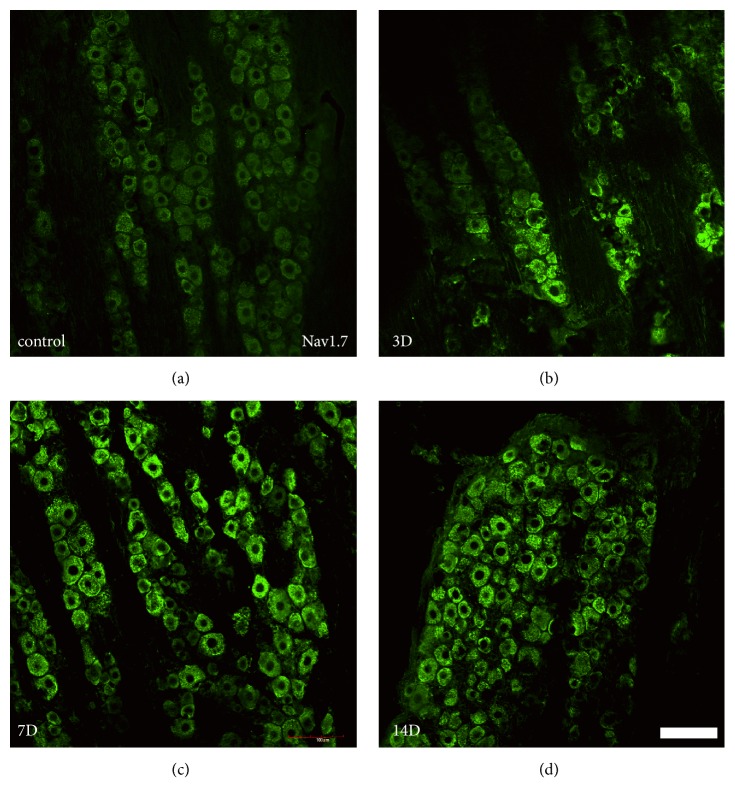
Expression of Nav1.7 in the TG pulpal afferent neurons. (a–d) Immunostaining showing Nav1.7 expression in the TG. The protein expression of Nav1.7 increased in the TG in a rat model of pulpitis from days 3 through 14. The highest level of protein expression was observed on day 14. Each group included four rats. Scale bar: 100 *μ*m.

**Figure 5:**
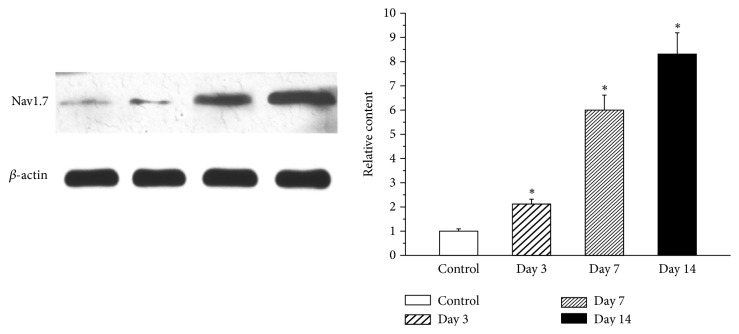
Western blot analysis of Nav1.7 expression in the TG of control and pulp-exposed rats. The protein expression of Nav1.7 increased in the TG from days 3 through 14 in a rat model of pulpitis. The highest level of protein expression was found on day 14. *∗* indicates a statistically significant difference (p <0.05) between the two groups. Each group included four rats.

To identify the levels of Nav1.7 present in the TG, protein expression levels of these two ion channels were examined in the TG using immunohistochemistry (Figure 4) and western blot analyses (Figure 5). Nav1.7 was expressed in the normal TG. Both the immunohistochemistry and western blot results showed an enhancement in the protein expression levels of Nav1.7 in the TG on days 3 through 14 after pulp exposure. The highest level of protein expression was observed on day 14.

### 3.4. Nav1.7 Inhibitor PF04856264 Reduced Inflammatory Pain

**Figure 6:**
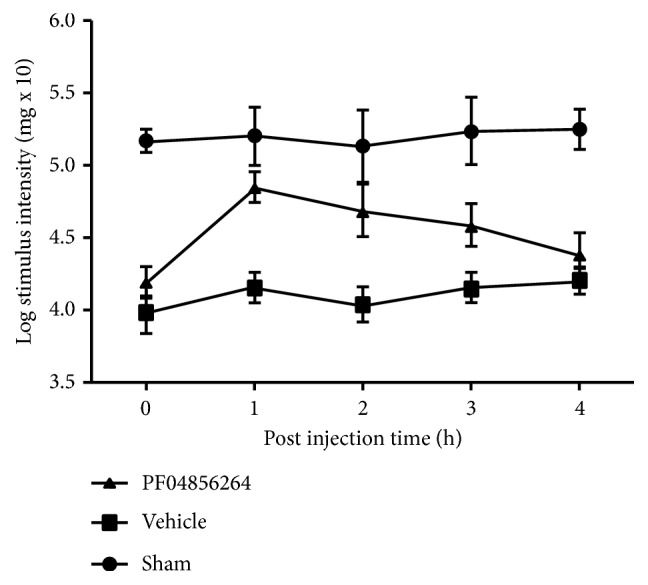
Effects of pulp exposure on mechanical hypersensitivity. The Nav1.7 in TG from the sham, pulpitis rats, and rats with pulpitis given PF04856264 (an Nav1.7 antagonist, 30 mg/kg) on postoperative day 14. PF04856264 significantly increased the facial mechanical pain threshold in the pulpitis rats (n=8, *∗∗*p<0.01). When PF04856264 was administered, the increase in threshold started at 0 h after and reached a maximum at 1h after injection.

The mechanical pain threshold was determined every hour after the tooth pulp exposure procedure surgery and given PF04856264 (a Nav1.7 antagonist, 30 mg/kg). There was a significant difference between the sham and the model in WTs within four hours after pulp exposure. The PF04856264 WTs, between the sham and the model, were increased and then decreased. An increase triggered by PF04856264 was noticed at 1h, where maximum WTs was measured, as compared with other hours. At 0 h and 4 h, there was no significant difference in the WT between PF04856264 and the model (Figure 6).

**Figure 7:**
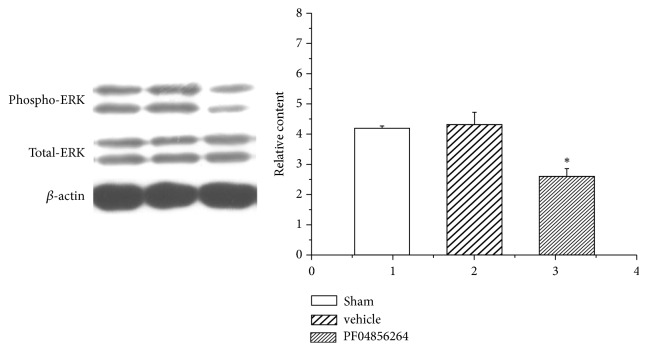
Western blotting analysis of ERK expression in the TG of control and pulp-exposed rats. The Nav1.7 in TG from the sham, pulpitis rats, and rats with pulpitis given PF04856264 (an Nav1.7 antagonist, 30 mg/kg) in the same blot on postoperative day 14. Representative gel images and summary data show that the intensity of ERK is increased in pulpitis rats compared with that in the sham rats.

The ERK in the TG from the sham rats, pulpitis rats, and rats with pulpitis given PF04856264 was determined by western blotting (Figure 7). Western blot results showed that the Nav1.7 inhibitor PF04856264 prohibited expression of ERK in the TG.

### 3.5. Expression of ERK in a Rat Model of Pulpitis

**Figure 8:**
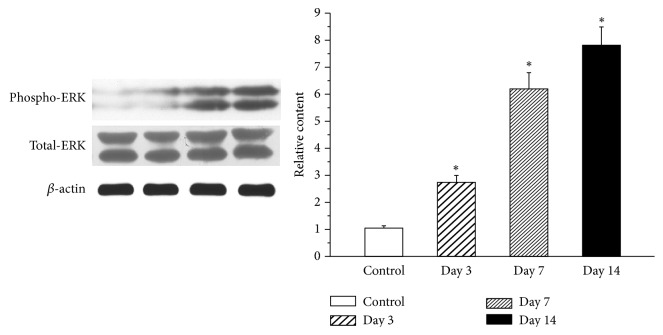
Western blot analysis of ERK expression in the TG of control and pulp-exposed rats. Western blot analysis of JNK expression in the TG in control and pulp-exposed rats. The protein expression of JNK increased in the TG from days 3 through 14 after pulp exposure. The highest level of protein expression was observed on day 14. *∗* indicates a statistically significant difference (p<0.05) between the two groups. Each group included four rats.

We examined whether pulpitis induces the upregulation of ERK in the TG by examining the protein levels of ERK on days 1, 7, and 14 after tooth pulp exposure. The production of ERK was significantly increased in the TG after pulp exposure and reached a peak on day 14 (Figure 8).

### 3.6. ERK Inhibitor PD98059 Prohibited Inflammatory Pain

**Figure 9:**
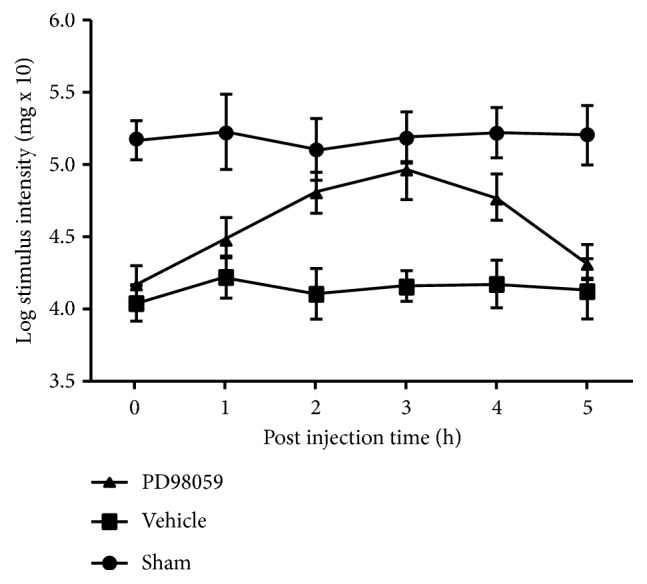
Effects of pulp exposure on mechanical hypersensitivity. The ERK in TG from the sham, pulpitis rats, and rats with pulpitis given PD98059 (an ERK antagonist, 10.0 *μ*M/L) on postoperative day 14. PD98059 significantly increased the facial mechanical pain threshold in the pulpitis rats (n=8, *∗∗*p<0.01). When PD98059 was administered, the increase of threshold started at 0 h and reached a maximum at 3h after injection.

The mechanical pain threshold was determined every hour after the tooth pulp exposure procedure and rats were given PD98059 (an ERK antagonist, 10.0 *μ*M/L). There was a significant difference between the sham and the model in WTs within 5 h after pulp exposure. The PD98059 WTs, between the sham and the model, increased and then decreased. An increase triggered by PD98059 was noticed at 3 h, where maximum WTs were measured compared with other hours. At 0h and 4h, there was no significant difference in WT between PD98059 and the model (Figure 9).

**Figure 10:**
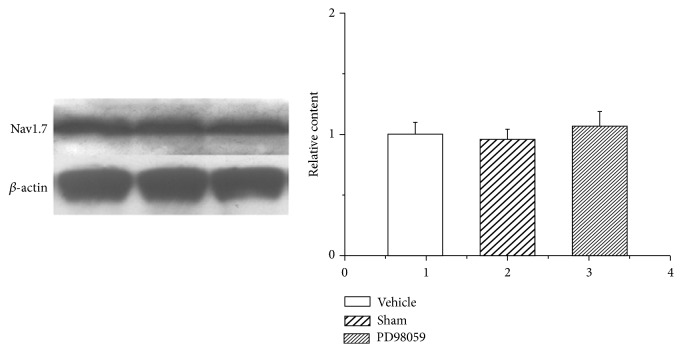
Western blot analysis of Nav1.7 expression in the TG of control and pulp-exposed rats. The ERK in TG from the sham, pulpitis rats, and those given PD98059 (an ERK antagonist, 10.0 *μ*M/L) in the same blot on postoperative day 14. Representative gel images and summary data show that representative gel images and summary data show no significant difference in Nav1.7 protein levels in pulpitis rats compared with the sham rats.

No significant difference in Nav1.7 protein levels was observed between sham rats, pulpitis rats and those given PD98059 in the TG (Figure 10).

Rats were used as animal models to study pulpitis at 3, 7, and 14 days, with dental pulp exposed for 14 days, except some were given PD98059 or PF04856264 and compared to sham groups. In this study, the pain threshold was significantly decreased compared with the sham group and pulp exposure increased rat reflexes to mechanical stimulation. We found that retrogradely labeled neurons were detected in the TG. We demonstrated that the reflex of rats to a mechanical stimulation increases after pulp exposure and that the exposed rat molar pulp can upregulate the expression of Nav1.7 in the rat TG. Furthermore, the JNK production in the TG increased in the rat model of pulpitis. With the established link between JNK, Nav1.7, and inflammatory pain, we identified its increased expression in tooth pulpitis. Extensive studies have investigated the role of ion channels and signaling mechanisms in inflammatory pain [22–24]. Recent studies found that voltage-gated sodium channels are involved in pain disorders. Involvement of trigeminal ganglionic Nav1.7 in hyperalgesia of inflamed temporomandibular joint is dependent on ERK1/2 phosphorylation [25].

There is recent interest in the role of VGSCs in chronic pain. Many chronic pain conditions exhibit the expression of Nav1.7 subtypes [26, 27]. Here we show that the expression levels of these two ion channels in the TG of rats were increased on days 3 through 14 after pulp exposure. Three of these channels (Nav1.7, Nav1.8, and Nav1.9) are expressed only in peripheral neurons [28]. Sensory neural excitability can be regulated by tetrodotoxin-sensitive (Nav1.7) and tetrodotoxin-insensitive (Nav1.8 and Nav1.9) channels [29]. Nav1.7 expression is increased in inflammatory pain [26, 30]. The mRNA expression levels of Nav1.7 are altered in the gingival tissue of patients with trigeminal neuralgia [22]. The ectopic activity or abnormal firing of afferent nerves after inflammation is hypothesized to be involved in the expression of Nav1.7 [27]. Studies have shown that the expression of the isoforms of Nav1.7 is increased in the dental pulp of painful teeth [31, 32].

The VGSCs contribute to neuronal hypersensitivity, particular in the inflammatory state [33]. Local anesthetic failure may be due to the upregulation of some VGSCs isoforms during the treatment of inflamed pulp [33–35]. In 1994, Yamasaki et al. established the pulpitis model used for pulp exposure [36]. These researchers found that pulpal necrosis extends gradually from the upper part of the pulpal tissue to the apex from days 1 through 14. Their results were consistent with our results in the TG. In this study, we investigated the expression of two Na channels, namely, Nav1.7 (TTS), in the TG over a period of several days after rat pulp exposure and found that the expression of these two channels increased from days 3 through 14 after pulp exposure, reaching a peak on day 14. There was a correlation between the expression levels of Nav1.7 and degree of inflammatory pain. In other words, higher expression levels of Nav1.7 were associated with a higher degree of inflammatory pain. Thus, we can infer that the degree of rat pulpitis pain induced by pulp exposure increased from days 3 through 14.

We then examined whether pulpitis induces the upregulation of ERK in the TG by examining the protein levels of pERK/ERK. In this study, we found that the expression of ERK increased from days 3 to 14 after pulp exposure and reached a peak on day 14. We confirmed that ERK is upregulated in TG with pulpitis. The MAPK family, which includes ERK, p38, and JNK, plays a significant role in the pathophysiological processes of inflammatory or neuropathic pain [11, 37, 38]. ERK pathway mediates orofacial neuropathic pain in the trigeminal ganglion of mice [39]. It has also been reported that ERK phosphorylation in trigeminal spinal subnucleus caudalis neurons is involved in pain associated with dry tongue [40]. The inhibition of MAPK pathways has been shown to attenuate inflammatory and neuropathic pain [9, 41]. Of these factors, JNK plays an important role in allodynia and chronic inflammatory diseases involving the expression of cytokines [42, 43]. JNKs can contribute to the expression of TNF-*α*, a major proinflammatory cytokine in rheumatoid arthritis. Its inhibitors, such as SP600125, protect mice from joint damage in rheumatoid arthritis animal models [42].

It has been shown that the phosphorylated forms of JNK can exaggerate pain sensation via the posttranslational modification of existing proteins and the transcriptional modulation of the expression of key genes in either peripheral nociceptors or second-order neurons [11, 37, 38]. Both Nav1.7 and ERK play an important role in inflammatory pain. These factors exhibited their highest production at the same time point. Thus, we can assume that there is an interaction between ERK and the Nav1.7 Na channels. The report shows that pERK1 phosphorylates specific residues within Nav1.7 and inhibition of pERK1/2 causes a fast inactivation of Nav1.7 without altering current density [12]. Thus, we inferred that ERK is associated with Nav1.7 in the TG in a pulpitis model. Tompkins et al. provide evidence that MEK/ERK via signaling enhance NaV1.7 VGSCs and neuronal excitability in guinea pig cardiac ganglia [44]. In addition, in DRG and hippocampal neurons, Na^+^ influx via Na^+^ channels regulate the phosphorylation and dephosphorylation of ERK [45]. This mechanism may be a novel regulatory mechanism of cell excitability, which may play crucial roles in various physiological and pathological states [46]. pERK1 in sensory neurons are tonically upregulated via Ras/ERK1/2 signaling [47].

## 4. Conclusions

There was a correlation between the expression levels of Nav1.7 and ERK and the degree of inflammatory pain [48]. In addition, Nav1.7 feedback regulates ERK expression. We found that the inhibition of Nav1.7 causes a decline of ERK expression. It is intriguing to note that the Nav1.7 protein level was upregulated with increased phosphorylation of ERK1/ERK2 [49]. However, to the best of our knowledge, previous studies have not investigated the relationship between the ERK pathway and the expression of Na channels in the TG neurons in a pulpitis model. We report in this study that there is consistency in the timing of the expression of ERK and Na channels (Nav1.7) in a rat model of pulpitis.

## Data Availability

The data used to support the findings of this study are available from the corresponding author upon request.
