# Corrigendum to “Nav1.7 via Promotion of ERK in the Trigeminal Ganglion Plays an Important Role in the Induction of Pulpitis Inflammatory Pain”

**DOI:** 10.1155/2022/9826502

**Published:** 2022-01-10

**Authors:** Shukai Sun, Jiaxing Sun, Wenkai Jiang, Wei Wang, Longxing Ni

**Affiliations:** ^1^State Key Laboratory of Military Stomatology & National Clinical Research Center for Oral Diseases & Shaanxi Key Laboratory of Stomatology, Department of Operative Dentistry & Endodontics, School of Stomatology, Fourth Military Medical University, No. 145 Western Changle Road, Xi'an, Shaanxi 710032, China; ^2^Department of Ophthalmology, Eye Institute of Chinese PLA, Xijing Hospital, Fourth Military Medical University, China

In the article titled “Nav1.7 via Promotion of ERK in the Trigeminal Ganglion Plays an Important Role in the Induction of Pulpitis Inflammatory Pain” [1], the name of the second author is misspelt and the corrected author name, Jiaxing Sun, is shown above.
